# Temporal Trends in Practice Patterns After Introduction of Pediatric Hypertension Guidelines in Canada

**DOI:** 10.1001/jamanetworkopen.2023.55239

**Published:** 2024-02-08

**Authors:** Michael Wu, Allison Dart, Leanne Kosowan, Smita Roychoudhury, Joycelyne E. Ewusie, Alexander Singer, Rahul Chanchlani

**Affiliations:** 1 Michael G. DeGroote School of Medicine, Hamilton, Ontario, Canada; 2 Department of Pediatric and Child Health, Rady Faculty of Health Sciences, University of Manitoba, Manitoba, Canada; 3 Department of Family Medicine, Rady Faculty of Health Sciences, University of Manitoba, Manitoba, Canada; 4 Department of Pediatrics, McMaster University, Hamilton, Ontario, Canada; 5 The Research Institute, St Joseph’s Healthcare Hamilton, Hamilton, Ontario, Canada; 6 Division of Pediatric Nephrology, Department of Pediatrics, McMaster University, Hamilton, Ontario, Canada; 7 Department of Health Research Methods Evidence and Impact, McMaster University, Hamilton, Ontario, Canada

## Abstract

**Importance:**

In 2016 and 2017, respectively, new Canadian and US guidelines for diagnosis and management of pediatric hypertension (HTN) were published. Six years after their publication, it is unknown whether the recommendations have led to changes in primary care practice patterns.

**Objectives:**

To determine whether HTN guidelines are associated with changes in practice patterns among primary care clinicians.

**Design, Setting, and Participants:**

This retrospective, multicenter, population-based cohort study was conducted across 3 phases: January 1, 2011, to December 31, 2015 (era 1), January 1, 2016, to December 31, 2017 (washout period), and January 1, 2018, to December 31, 2019 (era 2). Data were collected from 7 Canadian provinces using the Canadian Primary Care Sentinel Surveillance Network Electronic Medical Record database. Eligible participants included children and adolescents (aged ≥3 to <18 years) with 1 or more encounters in the database. Data analysis was conducted from February 2022 to February 2023.

**Exposure:**

Implementation of the 2016 Hypertension Canada and 2017 American Academy of Pediatrics guidelines.

**Main Outcomes and Measures:**

The primary outcomes were annual BP screening documentation, high BP follow-up documentation at 6 months and 1-year, HTN prevalence, laboratory testing rates, and medication prescription rates. Interrupted time series analysis was used to assess the association of the introduction of the Canadian and US guidelines with outcomes.

**Results:**

The study included 343 191 children and adolescents (mean [SD] age at first encounter, 6.7 (4.6) years; 173 290 female [50.5%]; 169 901 male [49.5%]), including 235 094 patients in era 1 and 193 473 patients in era 2. In era 1, 55 550 patients (23.6%) had at least 1 BP measurement, and in era 2, 45 006 patients (23.3%) had at least 1 BP measurement. There was a significant increase in BP screening in era 2 from 26 876 of 148 554 screenings (18.1%) to 28 556 of 141 192 screenings (20.2%; β = 0.202; 95% CI, 0.009 to 0.390; *P* = .04), and the increasing trend was sustained. There was a significant decrease in the trend of follow-up of high BP measurement at 6 months (1265 of 4941 patients with BP measurements [25.6%] to 1718 of 7321 patients with BP measurements [23.5%]; β = −0.490; 95% CI, −0.758 to −0.223; *P* = .001) and 1 year (1974 of 4941 measurements [40.0%] to 2314 of 7321 measurements [31.6%]; β = −1.392; 95% CI, −1.573 to −1.212; *P* < .001) in era 2. The proportion of patients meeting HTN criteria significantly increased from 2540 of 55 550 patients (4.6%) in era 1 to 5690 of 45 006 patients (12.6%) in era 2 (β = 0.0210; 95% CI, 0.0021 to 0.0410; *P* = .03). There was no significant change in the trend of laboratory testing rates in era 2 (949 of 4941 patients tested [19.2%] to 1149 of 7321 patients tested [15.7%]; β = −0.159; 95% CI, −0.364 to 0.046; *P* = .12). The trend in prescribing of medications to patients with HTN also decreased in era 2 (1305 of 4941 patients prescribed medication [26.4%] to 1415 of 7321 patients prescribed medication [19.3%]; β = −0.605; 95% CI, −0.830 to −0.358; *P* < .001).

**Conclusions and Relevance:**

The findings of this cohort study within the Canadian primary care setting suggest that there was a significant increase in BP screening and HTN prevalence after the publication of national and international HTN guidelines; however, the follow-up of high BP was still suboptimal. Increasing rates of pediatric HTN emphasize the need for better adherence to pediatric HTN guidelines to improve care and outcomes.

## Key Points

**Question:**

Have primary care practice patterns changed after the publication of the 2016 Hypertension Canada and 2017 American Academy of Pediatrics guidelines for pediatric hypertension?

**Findings:**

In this cohort study of 343 191 children and adolescents in the Canadian primary care setting using interrupted time-series analysis, there was a significant increase in blood pressure screenings and prevalence of hypertension after the publication of the guidelines. However, the follow-up of high blood pressure was still suboptimal.

**Meaning:**

These findings suggest that the uptake of pediatric hypertension guidelines among primary care clinicians could be improved, specifically, regarding timely and consistent follow-up of high blood pressure among children and adolescents.

## Teaser

This cohort study analyzes changes in primary care practice in Canada after publication of guidelines for pediatric hypertension.

## Introduction

The prevalence of pediatric hypertension (HTN) has increased in recent decades and is associated with increasing rates of childhood obesity.^1^ There is substantial evidence that pediatric HTN tracks into adulthood and is associated with target organ damage.^2,3,4,5^ Despite the rising prevalence, pediatric HTN is frequently underdiagnosed and inadequately treated.^6^ This is partly due to inconsistent and conflicting recommendations by clinical practice guidelines and society statements regarding regular pediatric blood pressure (BP) screening.^7,8^ Moreover, children in primary care settings with newly diagnosed HTN rarely have secondary causes as compared with those receiving care from specialists.^9^

Canadian and US guidelines regarding diagnosis and management of pediatric HTN were introduced in 2016 and 2017, respectively, by Hypertension Canada^10^ and the American Academy of Pediatrics (AAP).^7^ The Hypertension Canada guidelines^10^ provided comprehensive recommendations specific to BP measurement and updated criteria for HTN diagnosis and investigations. One notable recommendation was that BP should be measured regularly in all children aged 3 years or older.^10^ Similarly, the AAP guidelines^7^ suggested annual BP screening for all children and adolescents between 3 and 18 years, and a BP screening at every health care visit for those with obesity, diabetes, chronic kidney disease (CKD), or aortic coarctation.^7^ AAP guidelines also redefined normative BP data, updated HTN cut-offs, and limited unnecessary secondary HTN investigations among children 6 years and older and those suspected of having primary HTN.^7^

The impact of these guidelines on clinical practice remains unclear. Six years after their publication, it is unknown whether the recommendations have led to changes in primary care practice. To this end, we performed interrupted time series analysis to assess the change in practice following the introduction of these guidelines, including temporal changes to BP screening and follow-up, HTN prevalence, and practice patterns (ie, HTN investigations and medication prescribing). We hypothesized that there would be significantly increased rates of BP screenings, follow-up, and prevalence of HTN, as well as an increase in relevant laboratory investigations and medication prescriptions after the guidelines’ introduction.

## Methods

### Study Design

We conducted a retrospective cohort study using electronic medical record (EMR) data from the Canadian Primary Care Sentinel Surveillance Network (CPCSSN), a pan-Canadian, EMR-based data repository.^11^ This study was approved by the Health Research Ethics Board at the University of Manitoba. Individual patient informed consent was not required for this study due to the use of secondary deidentified data in accordance with the Common Rule. The study followed the Strengthening the Reporting of Observational Studies in Epidemiology (STROBE) reporting guideline for cohort studies.

### Setting and Data Source

CPCSSN contains deidentified, primary care EMR data from 1574 primary care family physicians, nurse practitioners, and community pediatricians representing approximately 1.5 million patients in 8 Canadian provinces (ie, British Columbia, Alberta, Manitoba, Ontario, Quebec, Nova Scotia, and Newfoundland and Labrador). CPCSSN amalgamates data from 11 different EMR vendors and 14 regional networks. All patients can opt out of being included in the CPCSSN database. All EMR data from consenting clinicians was included unless a patient opted out. The CPCSSN database represents approximately 4% of the Canadian population and approximately 8% of Canadian children. When sex-adjusted and age-adjusted, CPCSSN patients are representative of the Canadian population.^12^ Furthermore, validity and accuracy of this data set has been assessed.^12^ This study assessed the billing, encounter diagnosis, health conditions, medications, physical examination, laboratory results, and patient and clinician characteristics. The primary outcomes were annual BP screening documentation, high BP follow-up documentation at 6 months and 1 year, HTN prevalence, laboratory testing, and medication prescription rates.

### Inclusion Criteria

Our study included all children and adolescents (≥3 to <18 years) that had at least 1 encounter with a primary care clinician participating in CPCSSN. The study was divided into 3 time periods to assess the outcomes before and after introduction of guidelines: January 1, 2011, to December 31, 2015 (era 1); January 1, 2016, to December 31, 2017 (washout period); and January 1, 2018, to December 31, 2019 (era 2).

### Exclusion Criteria

We excluded all encounters provided to children less than 3 years of age because routine BP screening is not recommended by existing guidelines for this age group. Additionally, we excluded all encounters among patients 18 years of age or older. Therefore, participants could age in or out of our study cohort. We excluded 984 children with CKD, the most common secondary cause for hypertension in children, which may influence BP screening and follow-up patterns. We also excluded the COVID-19 pandemic era (2020 and onward) due to substantial changes to in-person visit rates.

### Outcomes Ascertainment

All outcomes were evaluated in the 3 time periods described above. The outcomes are as follows:

1. BP screening and follow-up
  1a. Screening: proportion of children and adolescents (≥ 3 to <18 years) with a BP documentation each year among patients that attended an appointment with their primary care clinician in the same year.
  1b. Follow-up: proportion of participants with a repeat BP measurement within 1 to 6 months and 1 month to 1 year after a high BP documented in the EMR. High BP was defined as BP at or above the 95th percentile for age, sex, and height (3-12 years) or greater than or equal to 130/80 mm Hg (≥13 years). Although the guidelines recommend follow-up within 2 weeks, we examined 6-month and 12-month follow-up as a practical application.
2. Prevalence of HTN: HTN was defined based on the era. The National Heart Lung and Blood Institute guideline^13^ was used to define thresholds for HTN (BP at or above the 95th percentile) up to December 31, 2017 (eTable 1 in Supplement 1). From January 1, 2018, hypertension was defined by the 2017 AAP guidelines^28^ as BP at or above the 95th percentile for age, sex, and height (3-12 years) or greater than or equal to130/80 mm Hg (≥13 years old). Height and weight percentile for each child was determined based on growth charts from the Centers for Disease Control and Prevention. Similar to other studies using the same data holding,^14,15^ HTN was defined as having at least 2 high BP measurements documented in the EMR.
3. Practice patterns of primary care clinicians: evaluated by laboratory results for creatinine, electrolytes, or lipid profile and antihypertensive prescriptions (eg, angiotensin-converting enzyme inhibitors, beta blockers, diuretics, and calcium channel blockers).

### Covariates

Patient-related covariates included age at first clinical encounter, age at HTN detection, sex, urban vs rural residence (defined using the first 3 characters of the postal code), social and material deprivation index, being overweight or obese, hyperlipidemia, and diabetes. The social and material deprivation index uses a postal code conversion file that links the patient’s 6-digit postal code to aggregate-level measures of social and material deprivation (educational level; employment-population ratio; mean personal income; proportion of persons living alone; proportion of individuals separated, divorced, or widowed; and proportion of single-parent families), dividing the population into quintiles from least deprived (score of 1) to most deprived (score of 5).^16^ Participants were considered overweight if their body mass index (BMI; calculated as weight in kilograms divided by height in meters squared) was above the 97th percentile (if ≤5 years) or above the 85th percentile (if >5 years).^17,18^ Hyperlipidemia was determined based on pediatric cutoffs for total cholesterol, low density lipoprotein C, non–high density lipoprotein C, triglycerides, and high-density lipoprotein C thresholds taken from National Heart, Lung, and Blood Institute guidelines.^13^ CPCSSN case definition identified patients with diabetes.^19^ CKD was defined based on an estimated glomerular filtration rate less than 90 mL/min/1.73 m^2^ based on the CKD Schwartz estimated glomerular filtration rate equation.^20^ Clinician-related covariates included sex, age, urban vs rural practice, province, and clinician type (eg, family physician, nurse practitioner, or pediatrician).

### Statistical Methods

We created annual cohorts of patients that met the study inclusion criteria and visited a primary care clinician each study year. Patient characteristics were described within each annual cohort using mean (SD), and frequency (percentage). Annual BP documentation among active patients (≥1 visit that year) was assessed. Active patients with 1 or more documented, high BP measurements were assessed for follow-up BP documentation within 6 months and 1 year, as well as laboratory orders and medications. We compared patients in the preguideline and postguideline eras using χ^2^ and *t* tests.

Interrupted time series analysis (ITS) was used to assess the association of the introduction of the Canadian (2016) and AAP (2017) guidelines with our outcomes of interest.^21,22^ The ITS analysis was performed by using segmented linear regression models to estimate change in level (immediate outcome) and change in trend (sustained or gradual outcome) in the outcomes between the preguideline and postguideline eras. Our regression models included 60 time points (months) for preguideline introduction (era 1) and 24 time points (months) for postguideline introduction (era 2). Due to the timing of the introduction of the guidelines, we treated the period between January 2016 and December 2017 as a washout period and excluded it from our analysis.^23^ The models examined the level of the outcome at baseline or preguideline (β0), a linear trend before the guideline (β1), a change in level of the outcome after the guideline (β2 [immediate outcome]), and a change in trend after the guideline (β3 [outcome over time or sustained outcome]).^24^ Beta (β) coefficients and corresponding 95% CIs and *P* values were reported. Furthermore, we inspected data points visually by plotting monthly data points and segmenting regression lines for each outcome of interest.

We tested for autocorrelation using the Durbin-Watson statistic and statistically significant, appropriate adjustment for autocorrelation was calculated.^22,25^ There is no defined rule on sample size requirements for an ITS design.^26,27^ Power depends on the number of data points, seasonal variation in data points, and heterogeneity of the data.^27^ Additionally, according to Wagner et al,^22^ a minimum of 24 monthly data points is required to accurately investigate the seasonal outcomes. We performed sensitivity analysis by reducing era 1 to 2 years (24 months; January 1, 2014, to December 31, 2015) to align it with era 2. Furthermore, we performed exploratory stratified analysis for BP screening and HTN diagnosis by BMI (overweight and obese vs normal BMI) and age (≤6 years and >6 years). Statistical analyses were conducted using SAS statistical software version 9.4 (SAS Institute). All statistical tests were 2-sided, and significance was defined as *P* value less than .05. Data analysis was conducted from February 2022 to February 2023.

## Results

### General Demographics

**Figure 1.**
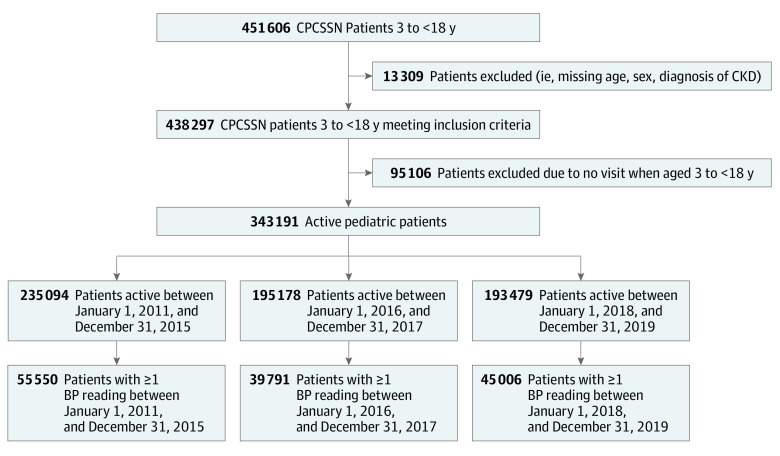
Flowchart of Study Population In this study, we defined active as having a pediatric visit during the year of analysis. Patients that had at least 1 pediatric visit during the study period were considered active. BP indicates blood pressure; CKD, chronic kidney disease; CPCSSN, Canadian Primary Care Sentinel Surveillance Network.

There were 343 191 active patients (173 290 female [50.5%]; 169 901 male [49.5%]) in our study after excluding by age, missing sex, and CKD (Figure 1). eTable 2 in Supplement 1 describes the annual patient population. The mean (SD) age of first encounter was 6.7 (4.6) years and the mean (SD) age at first BP measurement was 11.6 (5.1) years (Table 1). From 2011 to 2019, 88 312 participants (25.7%) had at least 1 BP measurement documented while 6553 (1.9%) met HTN criteria. Among those who had BP measurement documented, 6553 (7.4%) met HTN criteria.

**Table 1.** Study Population Demographics Based on Era

Characteristic	Patients, No. (%)			
	All years (N = 343 191)	Era 1: January 1, 2011-December 31, 2015 (n = 235 094)	Guideline introduction: January 1, 2016-December 31, 2017 (n = 195 178)	Era 2: January 1, 2018-December 31, 2019 (n = 193 473)
Age, mean (SD) y				
At first encounter,	6.7 (4.6)	7.5 (4.9)	5.5 (3.4)	4.9 (2.7)
At first BP reading	11.6 (5.1)	11.5 (5.2)	10.4 (5.0)	10.1 (4.8)
Sex				
Female	173 290 (50.5)	118 531 (50.4)	98 533 (50.5)	97 826 (50.6)
Male	169 901 (49.5)	116 563 (49.6)	96 645 (49.5)	95 647 (49.4)
Urbanicity				
Urban	275 549 (80.3)	189 804 (80.7)	158 466 (81.2)	155 984 (80.6)
Rural	67 642 (19.7)	45 290 (19.3)	36 712 (18.8)	37 489 (19.4)
Overweight or obese	62 583 (29.4)	46 899 (31.3)	43 124 (30.5)	43 239 (30.0)
Diabetes	2303 (0.7)	1824 (0.8)	1259 (0.7)	1101 (0.6)
Hyperlipidemia	2782 (0.8)	2292 (1.0)	1824 (0.9)	1666 (0.9)
Deprivation index, quintile^a^				
5	53881 (17.0)	37 059 (16.9)	30 999 (17.0)	29 650 (16.6)
4	57768 (18.2)	39 126 (17.8)	33 112 (18.2)	33 450 (18.7)
3	69095 (21.8)	44 935 (20.5)	37 762 (20.8)	40 221 (22.5)
2	72818 (23.0)	52 965 (24.1)	43 423 (23.9)	40 840 (22.8)
1	63112 (19.9)	45 567 (20.8)	36 612 (20.1)	34 836 (19.5)
BP documentation	88 312 (25.7)	55 550 (23.6)	39 791 (20.4)	45 006 (23.3)
Hypertension^b^	6553 (1.9)	2540 (1.1)	1988 (1.0)	5690 (2.9)
Clinician sex				
Female	188 273 (54.9)	125 205 (53.3)	109 152 (55.9)	108 486 (56.1)
Male	154 918 (45.1)	109 889 (46.7)	86 026 (44.1)	84 987 (43.9)
Clinician age, mean (SD) y	49.3 (9.7)	50.1 (9.5)	48.8 (9.6)	48.6 (9.7)
Clinic urbanicity				
Urban	319 035 (93.0)	218 711 (93.0)	180 920 (92.7)	179 146 (92.6)
Rural				
Clinician type				
Family physician	308 855 (90.0)	214 641 (91.3)	177 151 (90.8)	174 081 (90.0)
Other^c^	34 336 (10.0)	20 453 (8.7)	18 027 (9.2)	19 392 (10.0)
Province				
Alberta	93 849 (27.3)	69 207 (29.4)	56 560 (29.0)	50 565 (26.1)
British Columbia	20 361 (5.9)	12 875 (5.5)	11 508 (5.9)	11 426 (5.9)
Manitoba	63 759 (18.6)	34 881 (14.8)	30 580 (15.7)	40 054 (20.7)
Newfoundland and Labrador	832 (0.2)	183 (0.1)	79 (<0.1)	712 (0.4)
Nova Scotia	13 046 (3.8)	10 377 (4.4)	7652 (3.9)	6857 (3.5)
Ontario	145 984 (42.5)	107 236 (45.6)	86 483 (44.3)	79 826 (41.3)
Quebec	5360 (1.6)	335 (0.1)	2316 (1.2)	4039 (2.1)

Abbreviation: BP, blood pressure.

^a^ Social and material deprivation index was not available for 26 517 patients (8%) in the study.

^b^ Hypertension was defined as 2 high BP readings.

^c^ Other included nurse practitioners and pediatricians.

### Trends in BP Screening

In era 1, out of 235 094 patients who were seen, 55 550 (23.6%) had at least 1 BP measurement documented (eFigure 1 in Supplement 1). During the guideline introduction period, 39 791 of 195 178 patients (20.4%) had at least 1 BP measurement documented. In era 2, 45 006 of 193 473 patients (23.3%) had at least 1 BP measurement documented (eFigure 2 in Supplement 1).

**Figure 2.**
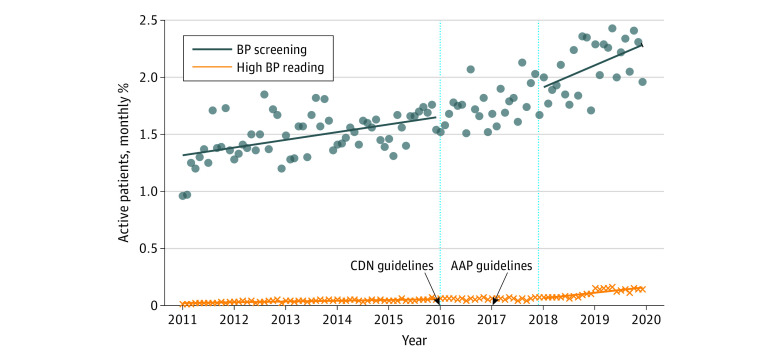
Blood Pressure Screening or Hypertension Documented in the Electronic Medical Record (EMR) The proportion of children who received an annual BP screening or had 2 or more high blood pressures documented in the EMR from January 1, 2011, to December 31, 2019, by a primary care clinician. The percentages are based on children with at least 1 BP measurement each year and as such, some children may have measurements done in more than 1 year. The line breaks between 2016 and 2018 represent the washout period, which is when the Canadian (CDN) and American Academy of Pediatrics (AAP) guidelines were introduced.

BP documentation increased each year. Among 90 684 patients who visited their primary care clinician in 2011, 12 051 (13.3%) had a BP documented in the EMR, and this increased to 28 556 of 141 192 patients (20.2%; *P* < .001) in 2019 (Figure 2 and eFigure 3 in Supplement 1). The proportion of BP screening increased following guideline introduction from 21 038 of 134 426 patients (15.7%) in 2015 to 26 876 of 148 554 patients (18.1%) in 2018. In addition, there was a significant increase in the level of BP screening post–guideline introduction from 26 876 of 148 554 patients (18.1%) to 28 556 of 141 192 patients (20.2%; β = 0.202; 95% CI, 0.009-0.390; *P* = .04). This increase was sustained throughout era 2 (β = 0.016; 95% CI, 0.004-0.028; *P* = .01) (Table 2 and Figure 2). In our sensitivity analysis, with a preguideline period of only 2 years (January 1, 2014, to December 31, 2015), we no longer saw an immediate increase in BP screening; however, we continued to see a significant sustained increase in BP screening in era 2 (eTable 3 in Supplement 1).

**Table 2.** Results of Interrupted Time-Series Analysis to Show the Association of Hypertension Guidelines With BP Screening, High BP Follow-Up, HTN Prevalence, Laboratory Follow-Up, and Medication Prescriptions

Measurement	β coefficient (95% CI)	*P* value
BP screening^a^		
Preguideline trend	0.006 (0.003 to 0.008)	<.001
Postguideline level change	0.202 (0.009 to 0.390)	.04
Postguideline trend change	0.016 (0.004 to 0.028)	.01
6-mo BP follow-up^b^		
Preguideline trend	0.116 (0.029 to 0.203)	.01
Postguideline level change	1.613 (−4.644 to 7.874)	.61
Postguideline trend change	−0.490 (−0.758 to −0.223)	.001
1-y BP follow-up^c^		
Preguideline trend	0.134 (0.047 to 0.221)	.003
Postguideline level change	−3.035 (−11.114 to 5.043)	.46
Postguideline trend change	−1.392 (−1.573 to −1.212)	<.001
Prevalence of HTN^d^		
Preguideline trend	0.0006 (0.0005 to 0.0007)	<.001
Postguideline level change	0.0210 (0.0021 to 0.0410)	.03
Postguideline trend change	0.0043 (0.0030 to 0.0050)	<.001
Laboratory follow-up of HTN^e^		
Preguideline trend	0.197 (0.153 to 0.241)	<.001
Postguideline level change	0.160 (0.130 to 0.180)	.12
Postguideline trend change	−0.159 (−0.364 to 0.046)	.12
Prescribing of medication to patients with pediatric HTN^f^		
Preguideline trend	0.240 (0.176 to 0.304)	<.001
Postguideline level change	2.717 (−2.841 to 8.276)	.33
Postguideline trend change	−0.605 (−0.830 to −0.358)	<.001

Abbreviations: ACF, first order autocorrelation; BP, blood pressure; DW, Durbin-Watson; HTN, hypertension.

^a^ DW = 1.840F; ACF = 0.050.

^b^ DW =0.894; ACF = 0.536.

^c^ DW = 0.676; ACF = 0.627.

^d^ DW = 0.606; ACF = 0.687.

^e^ DW = 1.291; ACF = 0.350.

^f^ DW = 0.898; ACF = 0.544.

In exploratory stratified analysis, there was no significant change in BP screening among patients based on BMI post–guideline introduction. (eTable 4, and eFigure 4 in Supplement 1). Participants older than 6 years saw an immediate significant increase in BP screening in era 2 (β = 0.92; 95% CI, 0.31 to 1.54; *P* = .004). Children 6 years and younger did not experience any significant change in level of BP screening in era 2 (β = −0.10; 95% CI, −0.41 to 0.21; *P* = .53) (eTable 4 and eFigure 5 in Supplement 1).

### Trend of High BP Follow-Up

BP follow-up after a high BP measurement was assessed for both 1 to 6 months and 1 month to 1 year time points. There were 19 781 participants (5.8%) with 1 or more high BP measurements documented, including 13 195 (5.6%) in era 1 and 14 903 (7.7%) in era 2. In era 1, 2382 patients with a high BP measurement (18.1%) had a follow-up BP measurement within 6 months and 2937 (22.3%) had a follow-up BP measurement within 1 year. In era 2, 2757 patients (18.5%) had a follow-up BP measurement within 6 months and 3894 (26.1%) had a follow-up BP measurement within 1 year.

Before the guideline introduction, there was an increasing trend in 6-month BP follow-up (316 of 2400 patients [13.2%] to 746 of 3466 patients [21.5%]; β = 0.116; 95% CI, 0.029 to 0.203; *P* = .01) and 1-year BP follow-up (657 of 2400 patients [27.4%] to 1227 of 3466 patients [35.4%]; β = 0.134; 95% CI, 0.047 to 0.221; *P* = .003). In era 2, there was a significant decrease in 6-month follow-up trends (1265 of 4941 patients [25.6%] to 1718 of 7321 patients [23.5%]; β = −0.490; 95% CI, −0.758 to −0.223; *P* = .001) and 1-year BP follow-up trends (1974 of 4941 patients [40.0%] to 2314 of 7321 patients [31.6%]; β = −1.392; 95% CI, −1.573 to −1.212; *P* < .001) (Table 2 and eFigure 6 in Supplement 1).

### Prevalence of HTN

In era 1, 2540 of 235 091 participants (1.1%) met HTN criteria with a mean (SD) onset age of 13.3 (4.1) years. Among the 55 550 patients who had undergone BP screening in era 1, 2540 (4.6%) had HTN. Among these participants, 1380 (63.2%) were overweight or obese and 107 (4.2%) were prescribed HTN medications. In era 2, 5690 of 193 473 participants (2.9%) met HTN criteria with a mean (SD) onset age of 13.2 (4.2) years. Among the 45 006 patients who had undergone BP screening in era 2, 5690 (12.6%) had HTN (β = 0.0210; 95% CI, 0.0021-0.0410; *P* = .03). Among these participants, 2937 (53.9%) were overweight or obese and 245 (4.3%) were prescribed HTN medication.

The proportion of participants with HTN increased from 187 of 12 051 participants (1.6%) in 2011 to 2342 of 28 556 participants (8.2%) in 2019 (*P* < .001) (eFigure 7 in Supplement 1). There was a significant increase in trend of HTN prevalence in era 1 (187 of 12 051 participants [1.6%] to 764 of 21 038 participants [3.6%]; β = 0.0006; 95% CI, 0.0005-0.0007; *P* < .001) and era 2 (1547 of 26 876 participants [5.0%] to 2342 of 28 556 participants [8.2%]; β = 0.0043; 95% CI, 0.0030-0.0050; *P* < .001) (Table 2 and Figure 2). Sensitivity analysis with a shorter preguideline period did not significantly change the results (eTable 3 in Supplement 1). Stratified analysis based on BMI and age also showed similar results of increasing HTN prevalence (eTable 5, eFigure 8, and eFigure 9 in Supplement 1).

### Trend of Laboratory Testing and Medications

Laboratory ordering following a high BP measurement increased from 147 of 2400 patients with laboratory testing ordered (6.1%) in 2011 to 949 of 4941 patients with laboratory testing ordered (19.2%) in 2018, with a decrease in 2019 to 1149 of 7321 orders (15.7%) (eFigure 10 in Supplement 1). Before the guideline introduction, there was an increasing trend in laboratory follow-up (147 of 2400 participants [6.1%] to 544 of 3466 participants [15.7%]; β = 0.197; 95% CI, 0.153 to 0.241; *P* < .001). There was no change in laboratory ordering in the postguideline period (β = 0.160; 95% CI, 0.130 to 0.180; *P* = .12) and no significant change in trend (β = −0.159; 95% CI, −0.364 to −0.046; *P* = .12) (eTable 3 in Supplement 1).

**Figure 3.**
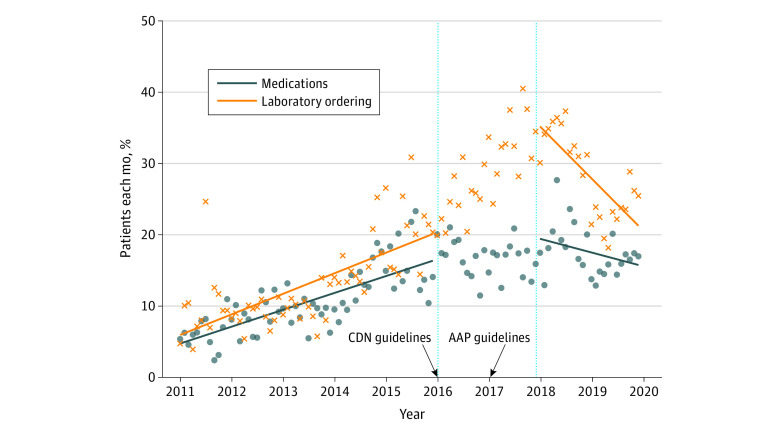
Documented Laboratory Follow-Up or Medication Prescription for Pediatric Patients That Had Hypertension The proportion of children who received medication prescriptions and laboratory testing from January 1, 2011, to December 31, 2019, by a primary care clinician. The line breaks between 2016 and 2018 represent the washout period, which is when the Canadian (CDN) and American Academy of Pediatrics (AAP) guidelines were introduced.

Prescriptions for antihypertensive medications among patients meeting HTN criteria increased from 282 of 2400 prescriptions (12.9%) in 2011 to 1305 of 4941 prescriptions (26.4%) in 2018 with a decrease in 2019 to 1415 of 7321 prescriptions (19.4%) (eFigure 10 in Supplement 1). Overall, there was increasing prescribing trend in era 1 (282 of 2400 [12.9%] to 700 of 3466 [20.2%]; β = 0.240; 95% CI, 0.176 to 0.241; *P* < .001); however, there was a significant decrease in prescribing trends in era 2 (1305 of 4941 [26.4%] to 1415 of 7321 [19.4%]; β = −0.605; 95% CI, −0.830 to −0.358; *P* < .001) (Table 2 and Figure 3).

## Discussion

In this cohort study of BP patterns in Canadian children and adolescents before and after the publication of revised pediatric hypertension guidelines, we saw a significant increase in BP screening and HTN prevalence. However, there was a significant decrease in follow-up of high BP at both the 6-month and 1-year time points after the guidelines were introduced. Furthermore, we did not observe a sustained change in ordering of relevant laboratory investigations, and there was a significant decrease in prescription patterns in the postguideline era.

Current guidelines^7^ recommend an annual BP screening among healthy children and adolescents aged 3 to 18 years. Our study demonstrated suboptimal adherence to these recommendations overall (only 23.3% of participants meeting criteria were screened); however, there was a promising increase (both immediate and sustained) in BP screenings after the introduction of these guidelines. A similar trend toward guideline adherence was identified with the decrease in the age of first BP documentation in era 2 with no change in the proportion of participants who were overweight or obese.

Similar to the published literature,^28,29^ we noticed a significant increase in the prevalence of HTN immediately after the guideline publication regardless of the BMI or age of the child, which was sustained throughout the study period. Several studies have evaluated the association of the AAP guidelines^30^ with the prevalence of hypertensive BP compared with the NHLBI guidelines.^13^ In 2019, Yang et al,^31^ in an international cohort of approximately 47 000 children, showed that prevalence of HTN was higher when children were classified based on AAP guidelines compared with the NHLBI guidelines. However, a major limitation of these studies is that HTN was defined by reclassifying the same cohort of children according to different guidelines and temporal trend of the impact of guidelines across time periods was not evaluated. Moreover, in some of these studies, BP was taken on a single day rather than multiple visits. We were able to address some of these knowledge gaps and demonstrated the outcomes of these guidelines in a pan-Canadian data set.

Both the Canadian and US guidelines^7,10^recommend follow up of an high BP measurement. Despite the increase in BP screening, we noticed a decreasing trend in the follow-up of high BP. This discrepancy between BP screening and follow-up of high BP has been shown in previous studies.^14^ Using the Toronto primary care EMR database, Aliarzadeh et al^32^ identified 5996 children and adolescents aged 3 to 18 years, of which 14% had at least 1 high BP measurement; of those particpants, only 5% had a follow-up measurement within 6 months. In that study,^32^ risk factors such as obesity and male sex were not associated with more frequent BP recording. Lower follow-up of high BP may be due to lack of clinician awareness regarding the timing of follow-up due to differences in training, or perhaps difficulty in accessing BP percentiles. In addition, it is likely that children with high initial BP readings were also followed by pediatric subspecialists in addition to their primary care clinician, which would decrease the primary care follow-up measurements. Ding et al^14^ had also assessed follow-up in this same data set and similarly encountered that follow-up BP measurements were also infrequently obtained for children with high BP readings and only 7.2% received timely follow-up within a month.

AAP guidelines^7^ also recommend limiting laboratory investigations among children who are older and suspected of having primary HTN. Our study showed a significant decrease in laboratory investigations immediately following the guideline publication, but the outcome was not sustained. However, it is unclear why medication prescriptions did not increase despite rising HTN prevalence. Notably, our study examined investigations and mediation patterns of patients after only 2 high BP readings were recorded rather than the 3 high BP readings necessary for a diagnosis of HTN.^14,15^ As such, some clinicians may have been waiting before initiating further workup, which may explain the lower rates of investigation and medication follow-up. Another potential reason is that these patients could have been treated with lifestyle counseling consistent with current recommendations^11^ or may have been referred to specialists for further treatment. Unfortunately, information regarding referrals and lifestyle counseling were unavailable and therefore not evaluated.^7^

We investigated the potential outcomes of national and international HTN guidelines on a diverse, pan-Canadian primary care population by leveraging high-quality EMR data. Another novel aspect is that we used ITS to evaluate the association of pediatric HTN guidelines with clinical practice. Importantly, we used 2 high BP measurements (rather than diagnostic codes) to define hypertension, which has been shown to underestimate HTN.^14,16^

### Limitations

There are some limitations of the study. First, era 2 had fewer years, and, consequently, fewer data points than era 1, which was related to the COVID-19 pandemic (2020-2022). However, we had adequate power to perform ITS.^22^ Moreover, we conducted a sensitivity analysis to assess implications of a shorter preguideline period and found similar results. However, due to reduced sample size, these results should be interpreted with caution. Second, ITS cannot establish causality; our results may be influenced by confounders not captured in analysis, such as salt intake, physical activity, environmental factors, and family history. Third, BP measurement technique was not documented, and it is uncertain what proportion of centers used auscultatory devices to measure BP. However, this variability should be present in both eras because measurement technique has not substantially changed across Canada. Fourth, primary care clinicians use the EMR to support their clinical practice, therefore data entry is not necessarily consistent. Because we captured BP measurements documented in the EMR’s coded fields, we may have missed those documented as a free-text or those not documented. Fifth, we defined HTN as 2 or more high BP readings instead of 3 or more high BP readings as recommended by guidelines to minimize the impact of BP documentation in the EMR on our study sample size. The use of 3 BP readings would have reduced the sample size by 37%. Sixth, although we describe representation from province and by clinician type, we did not control for province, practice, or clinician in our models.

## Conclusions

Consistent with the introduction of Canadian and US pediatric HTN guidelines,^7,10^ in this cohort study, we noticed a significant increase in BP screening and HTN prevalence. In addition, there was a reduction in prescription of antihypertensive medications after the guideline introduction. These findings suggest that the uptake of pediatric HTN guidelines among primary care clinicians could be improved through more consistent follow-up after high BP readings. As pediatric HTN is increasingly recognized, there is a greater need for adherence to pediatric hypertension guidelines to improve care and outcomes.

## Notes

Supplement 1. **eTable 1.** Classification of Office-Based BP in Children and Adolescents by the Fourth Report 2005, American Academy of Pediatrics 2017, and Hypertension Canada 2020 Guidelines **eTable 2.** Study Population Demographics Split Based on Individual Years **eFigure 1.** Preguideline Flow Diagram **eFigure 2.** Postguideline Flow Diagram **eFigure 3.** The Proportion of Children Who Receive an Annual Blood Pressure Screening From January 1, 2011, to December 31, 2019, by a Primary Care Provider **eTable 3.** Results of Interrupted Time-Series Analysis to Show the Impact of Hypertension Guidelines on BP Screening, Abnormal BP Follow-Up, HTN Prevalence and Laboratory and Medication Prescriptions, January 1, 2014-December 31, 2019 **eTable 4.** Results of Stratified Interrupted Time-Series Analysis to Show the Impact of Hypertension Guidelines on BP Screening by Children’s BMI and Age **eFigure 4.** Pediatric Patients With Blood Pressure Screening Documentation in the EMR of Primary Care Providers Participating in CPCSSN Between January 1, 2011, and December 31, 2019, by BMI **eFigure 5.** Pediatric Patients With Blood Pressure Screening Documentation in the EMR of Primary Care Providers Participating in CPCSSN Between January 1, 2011, and December 31, 2019, by Age **eFigure 6.** Pediatric Patients That Had a BP Documented With A Follow-Up Blood Pressure Screening Documented in the EMR of Primary Care Providers Participating in CPCSSN Between January 1, 2011, and December 31, 2019 **eFigure 7. **The Proportion of Children With a BP Screening Who Met HTN Criteria From January 1, 2011, to December 31, 2019, by a Primary Care Provider **eTable 5.** Results of Stratified Interrupted Time-Series Analysis to Show the Impact of Hypertension Guidelines on HTN Prevalence by Patient BMI, and Age **eFigure 8.** Pediatric Patients That Had a BP Screen With ≥2 Elevated Blood Pressures Documented in the EMR of Primary Care Providers Participating in CPCSSN Between January 1, 2011, and December 31, 2019, by BMI **eFigure 9.** Pediatric Patients With ≥2 Elevated Blood Pressures Documented in the EMR of Primary Care Providers Participating in CPCSSN Between January 1, 2011, and December 31, 2019, by Age **eFigure 10.** The Proportion of Children Who Underwent Laboratory Testing or Had a Medication Follow-Up From January 1, 2011, to December 31, 2019, by a Primary Care Provider

Supplement 2.
Data Sharing Statement
