# Supplementary material for: Temporal Trends in Practice Patterns After Introduction of Pediatric Hypertension Guidelines in Canada
Source: JAMA Netw Open. 2024 Feb 8;7(2):e2355239. doi: 10.1001/jamanetworkopen.2023.55239 (PMC10853834; doi:10.1001/jamanetworkopen.2023.55239)
Supplement: Supplement 1. — eTable 1. Classification of Office-Based BP in Children and Adolescents by the Fourth Report 2005, American Academy of Pediatrics 2017, and Hypertension Canada 2020 Guidelines eTable 2. Study Population Demographics Split Based on Individual Years eFigure 1. Preguideline Flow Diagram eFigure 2. Postguideline Flow Diagram eFigure 3. The Proportion of Children Who Receive an Annual Blood Pressure Screening From January 1, 2011, to December 31, 2019, by a Primary Care Provider eTable 3. Results of Interrupted Time-Series Analysis to Show the Impact of Hypertension Guidelines on BP Screening, Abnormal BP Follow-Up, HTN Prevalence and Laboratory and Medication Prescriptions, January 1, 2014-December 31, 2019 eTable 4. Results of Stratified Interrupted Time-Series Analysis to Show the Impact of Hypertension Guidelines on BP Screening by Children’s BMI and Age eFigure 4. Pediatric Patients With Blood Pressure Screening Documentation in the EMR of Primary Care Providers Participating in CPCSSN Between January 1, 2011, and December 31, 2019, by BMI eFigure 5. Pediatric Patients With Blood Pressure Screening Documentation in the EMR of Primary Care Providers Participating in CPCSSN Between January 1, 2011, and December 31, 2019, by Age eFigure 6. Pediatric Patients That Had a BP Documented With A Follow-Up Blood Pressure Screening Documented in the EMR of Primary Care Providers Participating in CPCSSN Between January 1, 2011, and December 31, 2019 eFigure 7. The Proportion of Children With a BP Screening Who Met HTN Criteria From January 1, 2011, to December 31, 2019, by a Primary Care Provider eTable 5. Results of Stratified Interrupted Time-Series Analysis to Show the Impact of Hypertension Guidelines on HTN Prevalence by Patient BMI, and Age eFigure 8. Pediatric Patients That Had a BP Screen With ≥2 Elevated Blood Pressures Documented in the EMR of Primary Care Providers Participating in CPCSSN Between January 1, 2011, and December 31, 2019, by BMI eFigure 9. Pediatric Pat [file jamanetwopen-e2355239-s001.pdf]

Supplemental Online Content

Wu M, Dart A, Kosowan L, et al. Temporal trends in practice patterns after introduction of pediatric hypertension guidelines in Canada. JAMA Netw Open. 2024;7(2):e2355239. doi:10.1001/jamanetworkopen.2023.55239

eTable 1. Classification of Office-Based BP in Children and Adolescents by the Fourth Report 2005, American Academy of Pediatrics 2017, and Hypertension Canada 2020 Guidelines
eTable 2. Study Population Demographics Split Based on Individual Years
eFigure 1. Preguideline Flow Diagram
eFigure 2. Postguideline Flow Diagram
eFigure 3. The Proportion of Children Who Receive an Annual Blood Pressure Screening From January 1, 2011 to December 31, 2019 by a Primary Care Provider
eTable 3. Results of Interrupted Time-Series Analysis to Show the Impact of Hypertension Guidelines on BP Screening, Abnormal BP Follow-Up, HTN Prevalence and Laboratory and Medication Prescriptions, January 1, 2014-December 31, 2019
eTable 4. Results of Stratified Interrupted Time-Series Analysis to Show the Impact of Hypertension Guidelines on BP Screening by Children's BMI and Age
eFigure 4. Pediatric Patients With Blood Pressure Screening Documentation in the EMR of Primary Care Providers Participating in CPCSSN Between January 1, 2011, and December 31, 2019, by BMI
eFigure 5. Pediatric Patients With Blood Pressure Screening Documentation in the EMR of Primary Care Providers Participating in CPCSSN Between January 1, 2011, and December 31, 2019, by Age
eFigure 6. Pediatric Patients That Had a BP Documented With A Follow-Up Blood Pressure Screening Documented in the EMR of Primary Care Providers Participating in CPCSSN Between January 1, 2011, and December 31, 2019
eFigure 7. The Proportion of Children With a BP Screening Who Met HTN Criteria From January 1, 2011 to December 31, 2019 by a Primary Care Provider
eTable 5. Results of Stratified Interrupted Time-Series Analysis to Show the Impact of Hypertension Guidelines on HTN Prevalence by Patient BMI and Age
eFigure 8. Pediatric Patients That Had a BP Screen With ≥2 Elevated Blood Pressures Documented in the EMR of Primary Care Providers Participating in CPCSSN Between January 1, 2011, and December 31, 2019, by BMI
eFigure 9. Pediatric Patients With ≥2 Elevated Blood Pressures Documented in the EMR of Primary Care Providers Participating in CPCSSN Between January 1, 2011, and December 31, 2019, by Age
eFigure 10. The Proportion of Children Who Underwent Laboratory Testing or Had a Medication Follow-Up From January 1, 2011 to December 31, 2019 by a Primary Care Provider

This supplemental material has been provided by the authors to give readers additional information about their work.

© 2024 Wu M et al. *JAMA Netw Open.*

eTable 1. Classification of Office-Based BP in Children and Adolescents by the Fourth Report 2005, American Academy of Pediatrics 2017, and Hypertension Canada 2020 Guidelines

**Guidelines**	**Fourth Report (2005)**	**American Academy of Pediatrics (2017)**	**Hypertension Canada (2016)**
BP screening and measurement	- Children 3 years and greater should have their BP measured whenever seen in a medical setting<br>-Children under age 3 should have BP measured when they have conditions such as known renal disease<br>- The preferred method of BP measurement is auscultatory approach, while measurements obtained by oscillometric approach that exceed the 90th percentile should be repeated by auscultation.<br><br>-ABPM only should be used by experts in field of pediatric HTN	- Annual BP measurement in children 3 years of age and greater<br>-BP measurement at every visit if child has risk factors for HTN<br>- Oscillometric approach can be used for screening, but the results must be confirmed by auscultatory approach<br>- Elevated BP should be confirmed on 3 separate clinic visits<br>- ABPM recommended	-BP should be regularly measured in children 3 years of age and older<br>-There is no recommendation on screening frequency<br>- Oscillometric approach can be used for screening, but the results must be confirmed by auscultatory approach<br>- Elevated BP should be confirmed on 3 separate clinic visits<br>- ABPM should be considered
Hypertensiion threshold	Blood pressure >95th percentile	≥95th percentile (<13 yr)<br>Or ≥130/80 (≥13 yr)	If either systolic or diastolic blood pressure is ≥95th Can be staged.<br>Stage 1 is 95th-99th percentile plus 5 mm Hg.<br>Stage 2 is BP >99th percentile plus 5 mm Hg
Target BP (general pediatric population)	<90th percentile	<90th percentile (<13 yr)<br>Or <130/80 (≥13 yr)	Not mentioned

© 2024 Wu M et al. *JAMA Netw Open.*

eTable 2. Study Population Demographics Split Based on Individual Years

	Total N= 343,191	2011 N= 90,684	2012 N= 105,909	2013 N= 115,647	2014 N= 128,009	2015 N= 134,355	2016 N= 141,657	2017 N= 147,653	2018 N= 148,445	2019 N= 141,058	2020 N= 108,148
Age (mean, SD)		8.0 (6.0)	8.1 (6.0)	8.1 (6.0)	8.1 (6.0)	8.1 (6.0)	8.2 (5.9)	8.3 (5.9)	9.0 (5.6)	9.7 (5.3)	10.4 (4.9)
Age of first clinical encounter (mean, SD)	6.7 (4.6)	8.6 (5.3)	8.0 (5.1)	7.3 (4.7)	6.7 (4.3)	6.2 (3.9)	5.7 (3.5)	5.2 (3.1)	5.0 (2.8)	4.7 (2.4)	4.4 (2.1)
Age First BP (mean, SD)	11.6 (5.1)	11.5 (5.0)	11.3 (5.1)	10.9 (5.1)	10.6 (5.1)	10.4 (5.0)	10.2 (5.0)	10.0 (4.9)	10.0 (4.8)	9.8 (4.7)	9.5 (4.6)
Female n(%)	173,290 (50.5)	46,401 (51.2)	53,955 (50.9)	58,788 (50.8)	64,747 (50.6)	68,195 (50.8)	71,861 (50.7)	74,996 (50.8)	75,285 (50.7)	71,993 (51.0)	55,803 (51.6)
Urban n(%)	275,549 (82.8)	74,312 (84.0)	86,574 (81.7)	94,873 (83.9)	104,297 (83.3)	109,237 (83.2)	115,378 (83.3)	120,354 (83.3)	120,179 (82.6)	114,333 (82.7)	18,344 (17.3)
Obesity n(%)	62,583 (29.4)	19,598 (33.8)	23,877 (33.5)	27,449 (33.2)	30,545 (32.6)	31,935 (31.8)	33,180 (31.2)	33,713 (30.7)	34,043 (30.4)	32,815 (30.3)	25,490 (30.8)

© 2024 Wu M et al. *JAMA Netw Open.*

Hyperlipidemia n(%)	2,782 (0.8)	1,140 (1.3)	1,279 (1.2)	1,408 (1.2)	1,451 (1.1)	1,517 (1.1)	1,453 (1.0)	1,470 (1.0)	1,410 (1.0)	1,275 (0.9)	963 (0.9)
Diabetes n(%)	1,514 (0.4)	593 (0.7)	619 (0.6)	684 (0.6)	742 (0.6)	767 (0.6)	782 (0.6)	793 (0.5)	769 (0.5)	679 (0.5)	655 (0.5)
Social and material deprivation index*(missing for 8% of the population)											
Q1 n(%)	63,112 (19.9)	19,736 (23.0)	21,478 (21.5)	23,040 (21.1)	25,105 (20.9)	26,332 (21.0)	27,182 (20.6)	27,775 (20.1)	26,912 (19.5)	25,687 (19.7)	19,833 (19.7)
Q2 n(%)	72,818 (23.0)	19,835 (23.1)	24,094 (24.1)	26,510 (24.3)	29,335 (24.4)	30,519 (24.3)	31,836 (24.1)	32,638 (23.7)	31,627 (23.0)	29,522 (22.6)	22,292 (22.6)
Q3 n(%)	69,095 (21.8)	16,901 (19.7)	19,591 (19.6)	21,771 (20.0)	24,178 (20.1)	25,399 (20.2)	26,905 (20.4)	28,577 (20.8)	30,590 (22.2)	29,196 (22.4)	22,528 (22.4)
Q4 n(%)	57,768 (18.2)	15,377 (17.9)	18,587 (18.6)	19,812 (18.2)	21,213 (17.7)	22,406 (17.8)	23,940 (18.1)	25,242 (18.3)	25,668 (18.6)	24,389 (18.7)	18,398 (18.7)
Q5 n(%)	53,881 (17.0)	13,937 16.3)	16,292 (16.3)	17,904 16.4)	20,235 (16.9)	20,947 (16.7)	22,349 (16.9)	23,505 (17.1)	22,912 (16.6)	21,639 (16.6)	16,442 (16.6)

© 2024 Wu M et al. *JAMA Netw Open.*

BP Documentation n(%)	125,217 (36.5)	12,569 (13.9)	16,292 (15.4)	18,640 (16.1)	20,058 (15.7)	22,648 (16.9)	25,388 (17.9)	28,090 (19.0)	26,911 (18.1)	28,923 (20.5)	14,097 (13.0)
BP Hypertension (2 abnormal BP)[L1]	5,931 (1.7)	187 (0.2)	412 (0.3)	530 (0.4)	647 (0.5)	764 (0.6)	960 (0.6)	1028 (0.6)	1547 (1.0)	2342 (1.7)	1137 (1.1)
BP classification: Hypertension (1 BP)	16,127 (4.7)	1,447 (1.6)	1,575 (1.5)	1834 (1.6)	2025 (1.6)	2,360 (1.8)	2,812 (2.0)	2,938 (2.0)	4669 (3.1)	5161 (3.7)	2665 (2.5)
BP classification: Prehypertension /elevated BP (1 BP)	13,881 (4.0)	1,201 (1.3)	1552 (1.5)	1736 (1.5)	1914 (1.5)	1992 (1.5)	2,292 (1.6)	2,521 (1.7)	3689 (2.5)	4259 (3.0)	1979 (1.8)
BP classification: Normal BP	95,209 (27.7)	9,921 (10.9)	13,165 (12.4)	15,070 (13.0)	16119 (12.6)	18,296 (13.6)	20,284 (1.4)	22,631 (15.3)	18,553 (12.5)	19,503 (13.8)	9453 (8.7)

© 2024 Wu M et al. *JAMA Netw Open.*

eFigure 1. Preguideline Flow Diagram

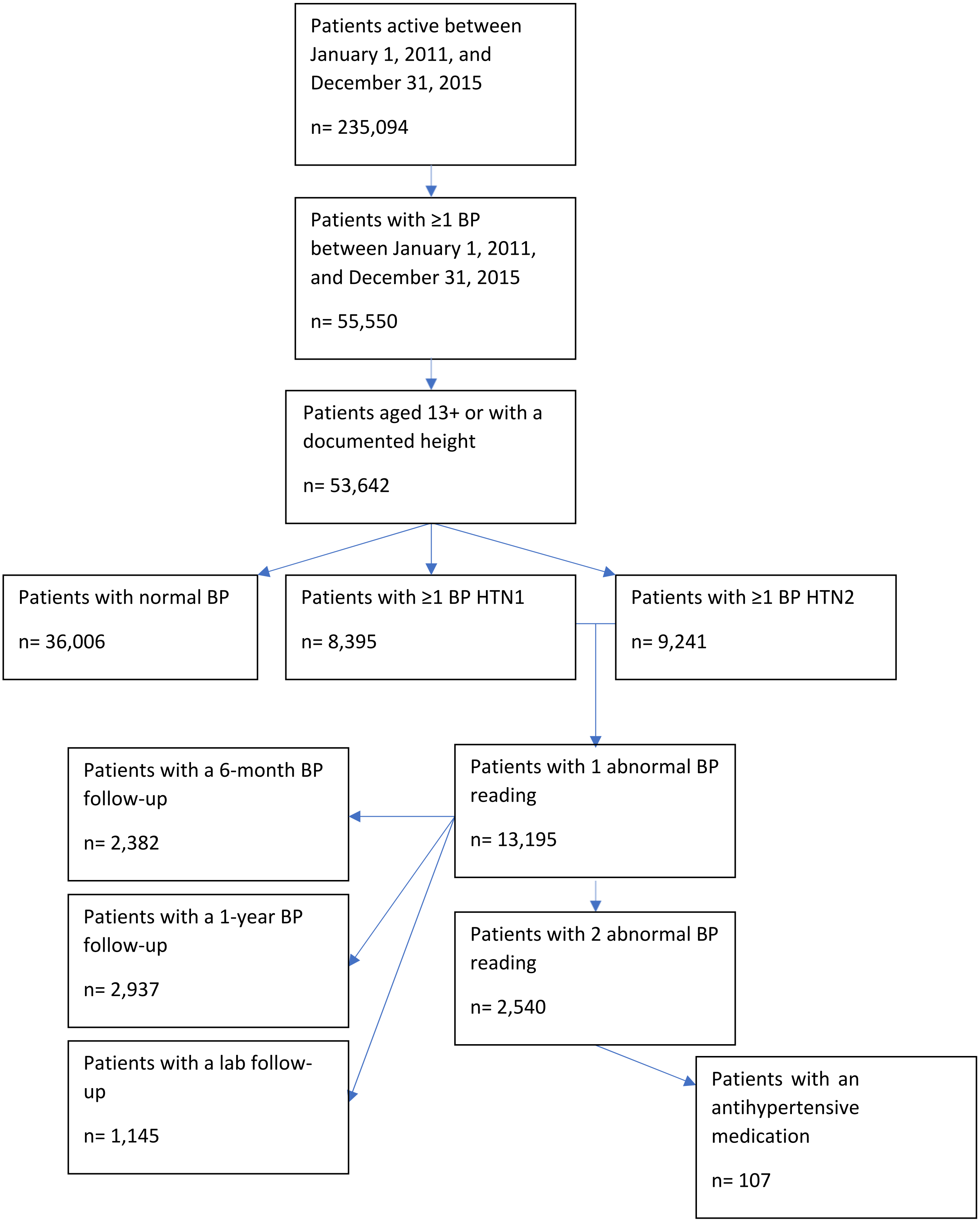

© 2024 Wu M et al. *JAMA Netw Open.*

eFigure 2. Postguideline Flow diagram.

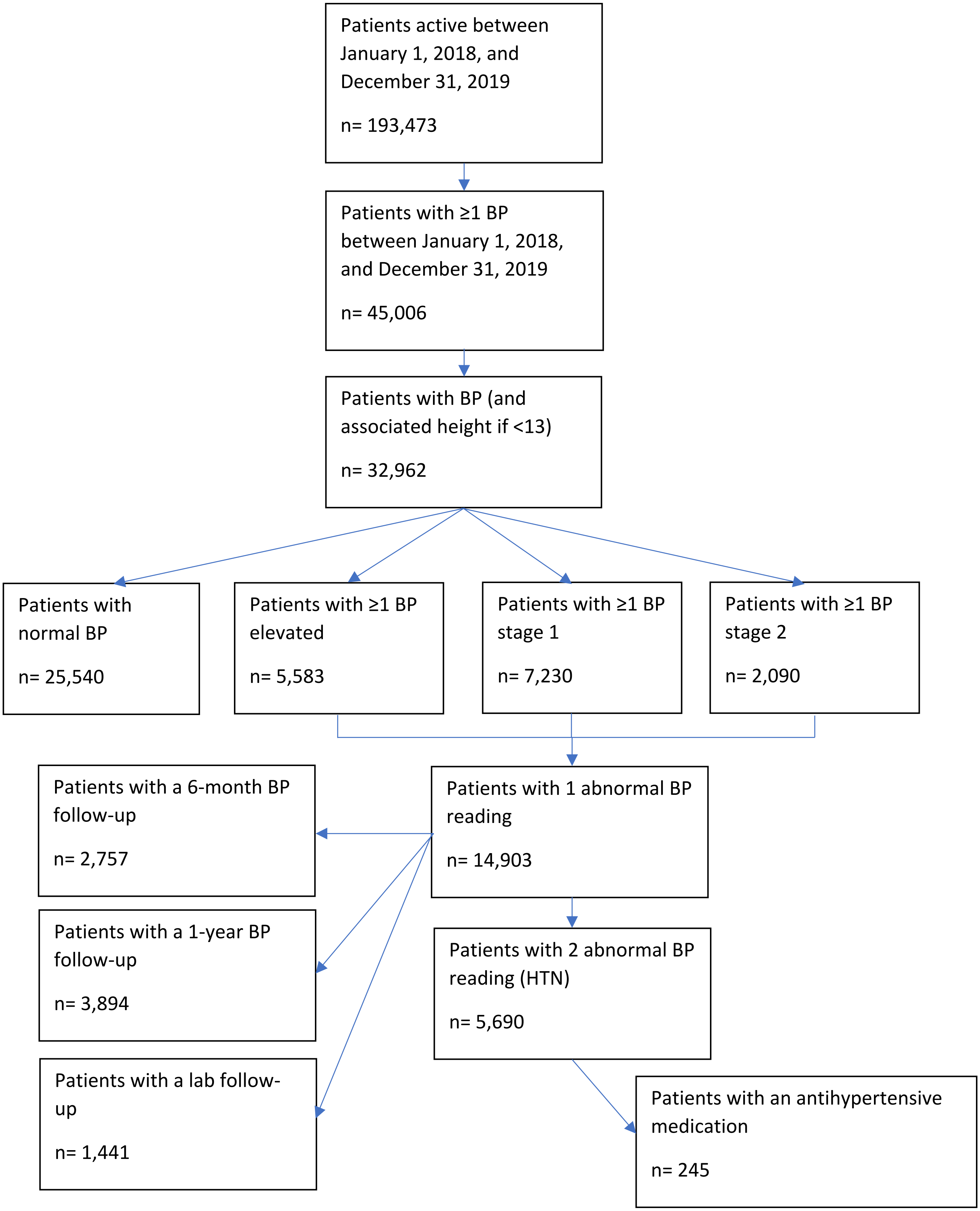

© 2024 Wu M et al. *JAMA Netw Open.*

eFigure 3. The Proportion of Children Who Receive an Annual Blood Pressure Screening From January 1, 2011 to December 31, 2019 by a Primary Care Provider

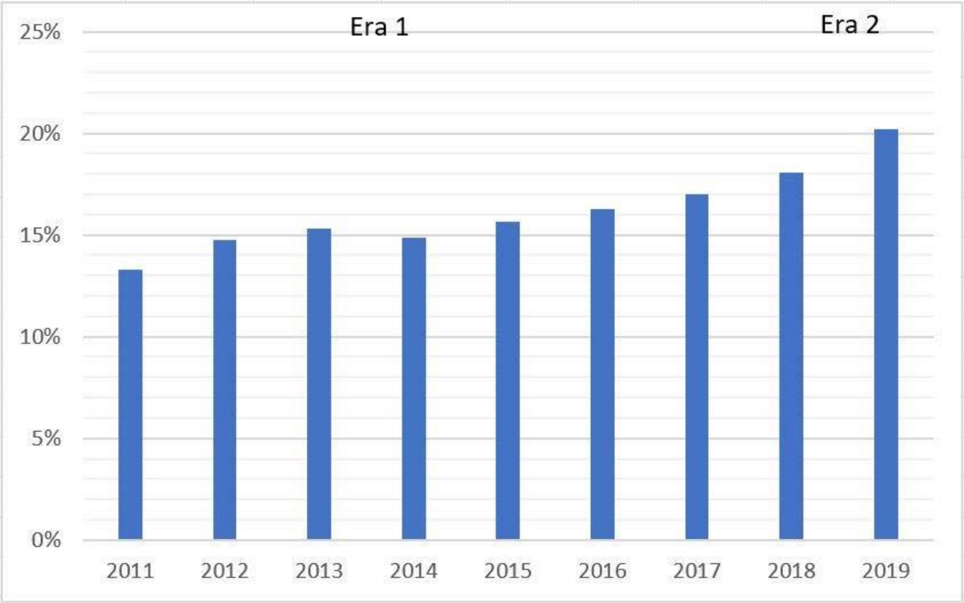

The percentages are based on children with at least 1 BP each year and as such, some children may have measurements done in more than 1 year.

© 2024 Wu M et al. *JAMA Netw Open.*

eTable 3. Results of Interrupted Time-Series Analysis to Show the Impact of Hypertension Guidelines on BP Screening, Abnormal BP Follow-Up, HTN Prevalence and Laboratory and Medication Prescriptions, January 1, 2014-December 31, 2019

	Beta Coefficient (95% CI)	P-value
**Monthly Blood Pressure Screening**		
Pre-guideline trend[1]	0.59 (0.005, 0.013)	0.0001
Guideline introduction	-0.98 (-0.41, 0.28)	0.7
Post-guideline trend[2]	0.5 (0.004, 0.28)	0.01
DW 2.397, ACF -0.234		
**6-month blood pressure follow-up**		
Pre-guideline trend[1]	0.07 (-0.13, 0.19)	0.707
Guideline introduction	1.37 (4.54, 25.23)	0.01
Post-guideline trend[2]	-0.63 (-0.76, -0.22)	0.001
DW 0.769, ACF 0.589		
**1-year blood pressure follow-up**		
Pre-guideline trend[1]	-0.28 (-0.63, 0.06)	0.1
Guideline introduction	1.86 (16.07, 43.35)	<.0001
Post-guideline trend[2]	-0.96 (-1.57, -1.21)	<.0001
DW 0.416, ACF 0.724		
**Monthly Prevalence of HTN**		
Pre-guideline trend[1]	0.58 (0.0003, 0.001)	0.0002
Guideline introduction	-0.56 (-0.09, -0.004)	0.034
Post-guideline trend[2]	0.84 (0.003, 0.01)	<.0001
DW 0.649, ACF 0.648		
**Laboratory follow-up of Hypertension**		
Pre-guideline trend[1]	0.44 (0.04, 0.27)	0.01
Guideline introduction	0.81 (-6.97, 8.91)	0.81
Post-guideline trend[2]	-0.32 (-0.36, -0.05)	0.12
DW 1.452, ACF 0.263		

© 2024 Wu M et al. *JAMA Netw Open.*

**Prescribing of medication to pediatric HTN patients**		
Pre-guideline trend[1]	0.71 (0.25, 0.5)	<.0001
Guideline introduction	1.04 (3.74, 26.7)	0.01
Post-guideline trend[2]	-0.74 (-0.85, -0.36)	<.0001
DW 0.792, ACF 0.592		

DW: Durbin-Watson D
ACF: 1st Order Autocorrelation
1: Pre-guideline period: January 1, 2014-December 31, 2015
2: Post-guideline period: January 1, 2018-December 31, 2019

© 2024 Wu M et al. *JAMA Netw Open.*

eTable 4. Results of Stratified Interrupted Time-Series Analysis to Show the Impact of Hypertension Guidelines on BP Screening by Children's BMI and Age

DW 1.832, ACF 0.044		
**Blood Pressure Screening by BMI** (BMI = overweight or obese)		
Pre-guideline trend	0.01 (-0.003, 0.03)	0.11
Post-guideline level change	-0.25 (-1.57, 1.07)	0.7
Post-guideline trend change	-0.01 (-0.05, 0.04)	0.8
DW 1.441, ACF 0.244		
**Blood Pressure Screening by BMI** (BMI = not overweight or obese)		
Pre-guideline trend	0.01 (0.001, 0.01)	0.02
Post-guideline level change	0.03 (-0.34, 0.4)	0.87
Post-guideline trend change	0.01 (0.01, 0.02)	0.02
DW 2.386, ACF -0.223		
**Blood Pressure Screening by age** (≤6 years)		
Pre-guideline trend	0.000 (-0.005, 0.01)	0.98
Post-guideline level change	-0.1 (-0.41, 0.21)	0.53
Post-guideline trend change	-0.004 (-0.01, 0.004)	0.33
DW 1.924, ACF -0.021		
**Blood Pressure Screening by age** (>6 years)		
Pre-guideline trend	-0.002 (-0.01, 0.01)	0.6
Post-guideline level change	0.92 (0.31, 1.54)	0.004
Post-guideline trend change	-0.02 (-0.04, 0.003)	0.1
DW 1.824, ACF 0.055		
DW: Durbin-Watson D<br>*ACF: 1st Order Autocorrelation*		

© 2024 Wu M et al. *JAMA Netw Open.*

© 2024 Wu M et al. *JAMA Netw Open.*

eFigure 4. Pediatric Patients With Blood Pressure Screening Documentation in the EMR of Primary Care Providers Participating in CPCSSN Between January 1, 2011, and December 31, 2019, by BMI

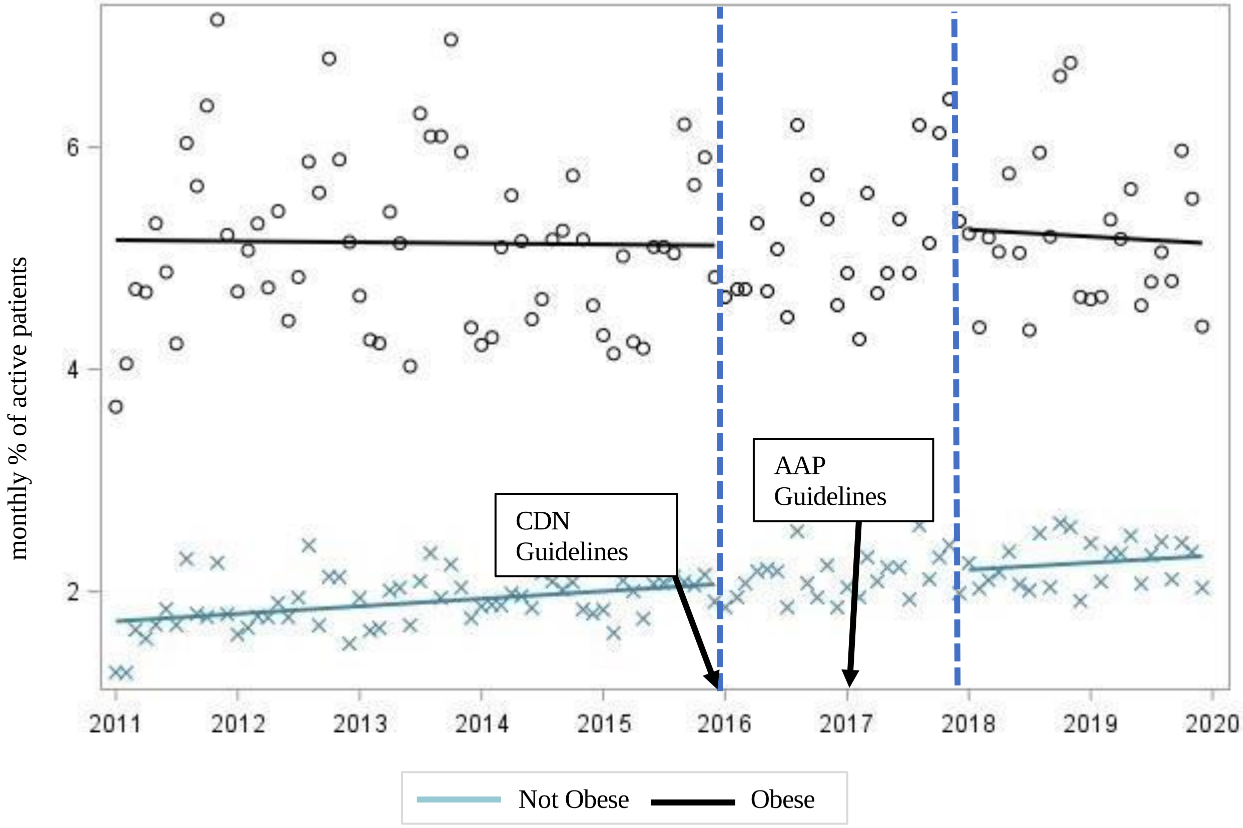

**CDN Guidelines: Hypertension Canada guidelines*
**AAP Guidelines: American Academy of Pediatrics Guidelines*

© 2024 Wu M et al. *JAMA Netw Open.*

eFigure 5. Pediatric Patients With Blood Pressure Screening Documentation in the EMR of Primary Care Providers Participating in CPCSSN Between January 1, 2011, and December 31, 2019, by Age

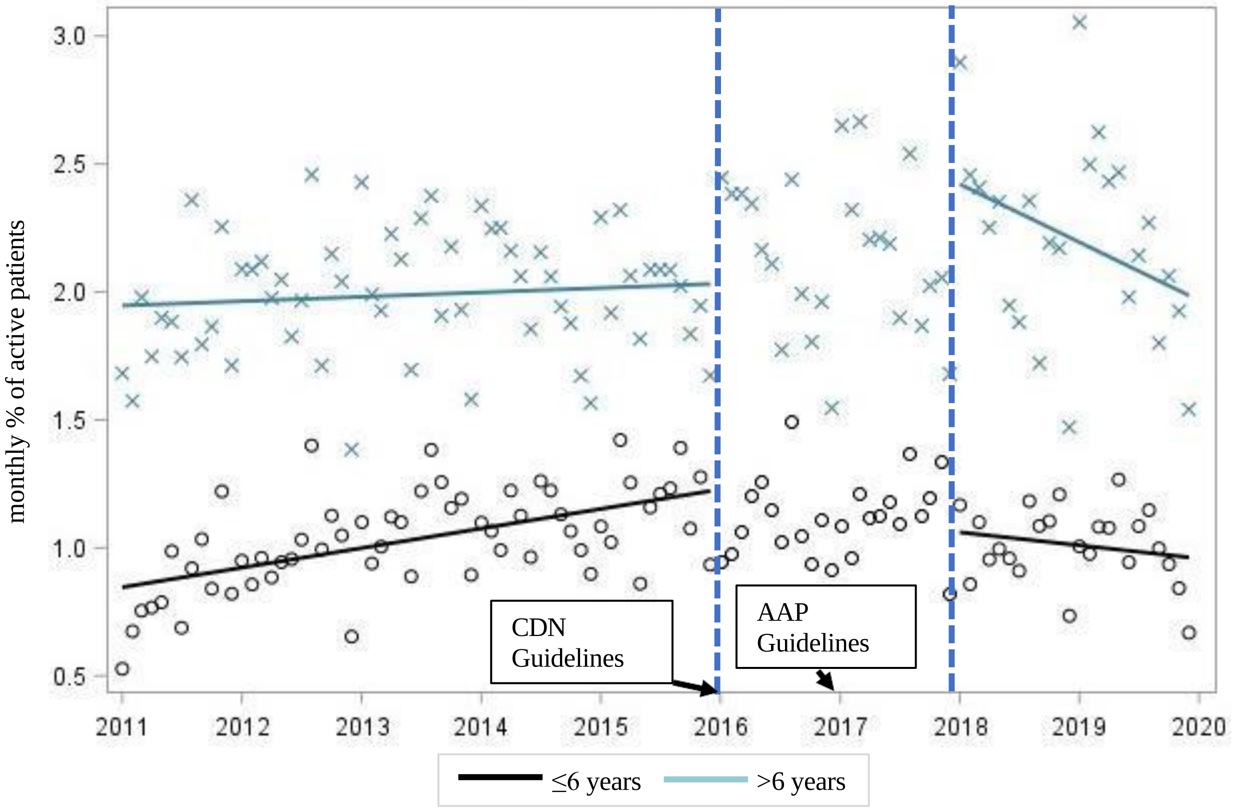

**CDN Guidelines: Hypertension Canada guidelines*
**AAP Guidelines: American Academy of Pediatrics Guidelines*

© 2024 Wu M et al. *JAMA Netw Open.*

eFigure 6. Pediatric Patients That Had a BP Documented With A Follow-Up Blood Pressure Screening Documented in the EMR of Primary Care Providers Participating in CPCSSN Between January 1, 2011, and December 31, 2019

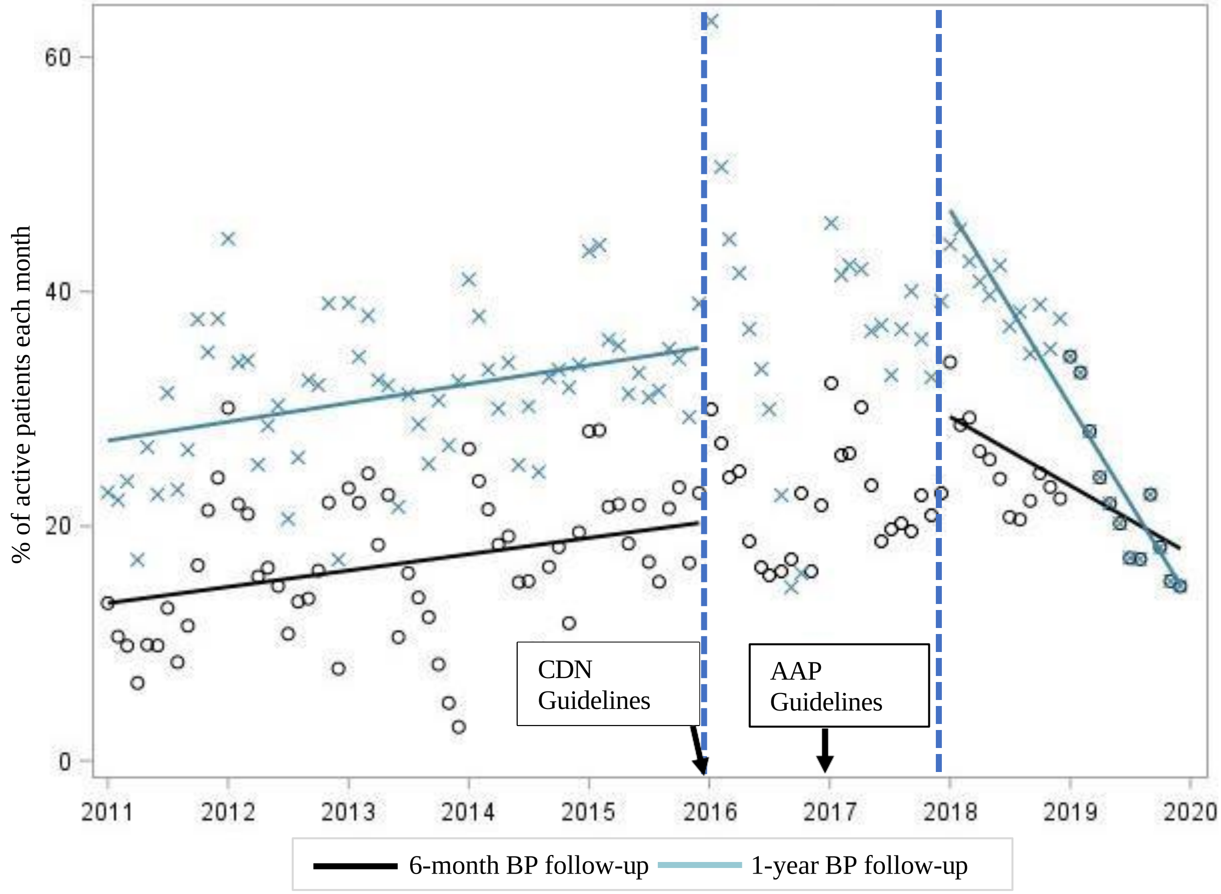

**CDN Guidelines: Hypertension Canada guidelines*
**AAP Guidelines: American Academy of Pediatrics Guidelines*

© 2024 Wu M et al. *JAMA Netw Open.*

eFigure 7. The Proportion of Children With a BP Screening Who Met HTN Criteria From January 1, 2011 to December 31, 2019 by a Primary Care Provider

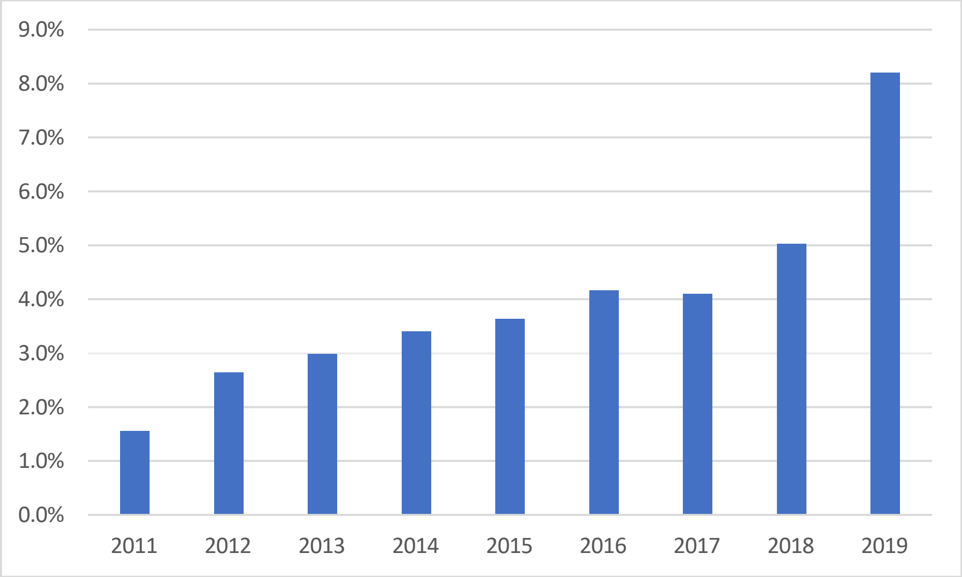

The NHLBI guideline was used to define thresholds for hypertension (blood pressure>95th percentile) up to December 31, 2017. From January 1, 2018, hypertension was defined by AAP 2017 guidelines as BP ≥95th percentile for age, sex and height [3-12 years] or ≥130/80mmHg [≥13 years]

© 2024 Wu M et al. *JAMA Netw Open.*

eTable 5. Results of Stratified Interrupted Time-Series Analysis to Show the Impact of Hypertension Guidelines on HTN Prevalence by Patient BMI and Age

**HTN by BMI** (BMI = overweight or obese)		
Pre-guideline trend	0.01 (0.01, 0.02)	<.001
Post-guideline level change	-0.04 (-0.32, 0.24)	0.79
Post-guideline trend change	0.03 (0.01, 0.05)	0.003
DW 1.616, ACF 0.186		
**HTN by BMI** (BMI = not overweight or obese)		
Pre-guideline trend	0.02 (0.02, 0.03)	<.001
Post-guideline level change	0.06 (-0.58, 0.7)	0.85
Post-guideline trend change	0.128 (0.09, 0.16)	<.001
DW 0.742, ACF 0.597		
**HTN by age** (≤6 years)		
Pre-guideline trend	0.001 (0.0004, 0.002)	0.001
Post-guideline level change	-1.97 (-3.64, -0.3)	0.02
Post-guideline trend change	0.17 (0.12, 0.22)	<.001
DW 1.565, ACF 0.696		
**HTN by age** (>6 years)		
Pre-guideline trend	0.02 (0.01, 0.02)	<.001
Post-guideline level change	1.06 (0.3, 1.83)	0.01
Post-guideline trend change	0.17 (0.12, 0.22)	<.001
DW 0.459, ACF 0.759		
DW: Durbin-Watson D<br>*ACF: 1st Order Autocorrelation*		

© 2024 Wu M et al. *JAMA Netw Open.*

eFigure 8. Pediatric Patients That Had a BP Screen With ≥2 Elevated Blood Pressures Documented in the EMR of Primary Care Providers Participating in CPCSSN Between January 1, 2011, and December 31, 2019, by BMI

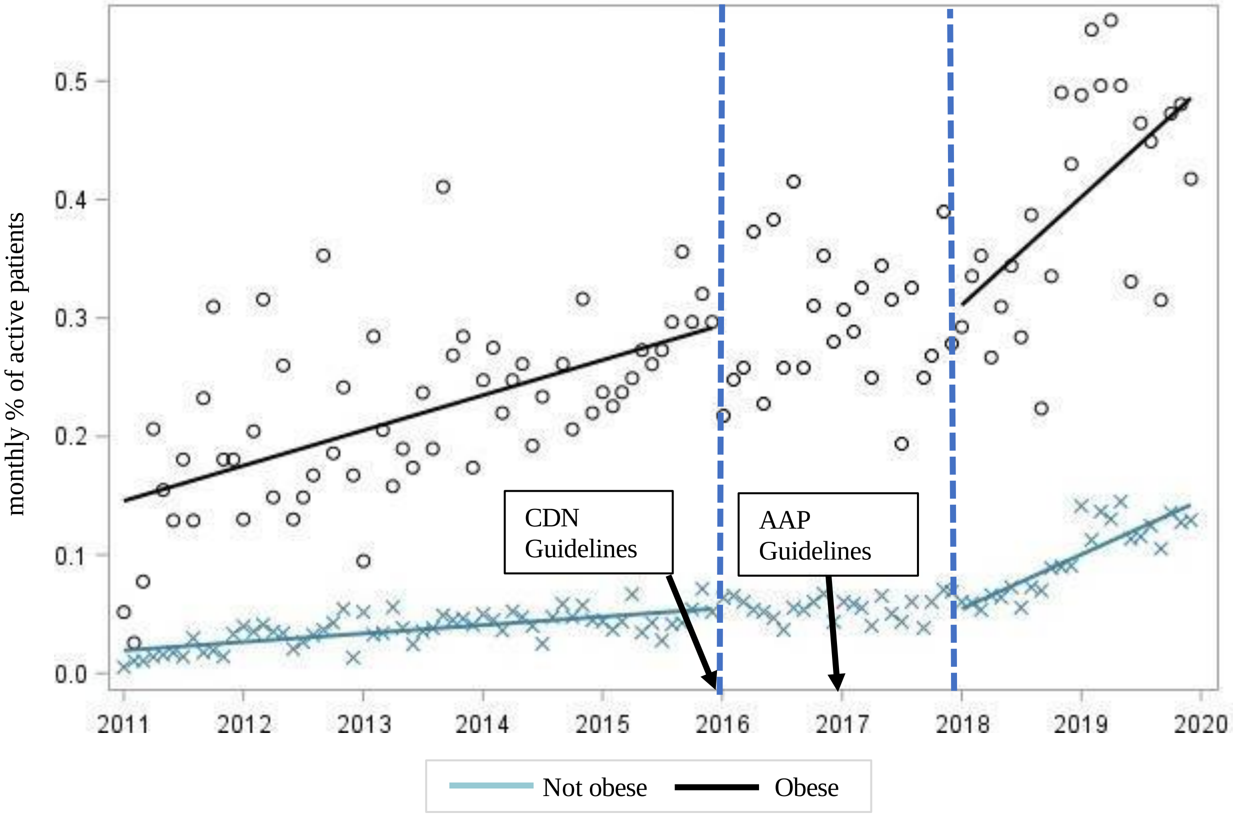

CDN Guidelines: Hypertension Canada guidelines
AAP Guidelines: American Academy of Pediatrics Guidelines

© 2024 Wu M et al. *JAMA Netw Open.*

eFigure 9. Pediatric Patients With ≥2 Elevated Blood Pressures Documented in the EMR of Primary Care Providers Participating in CPCSSN Between January 1, 2011, and December 31, 2019, by Age

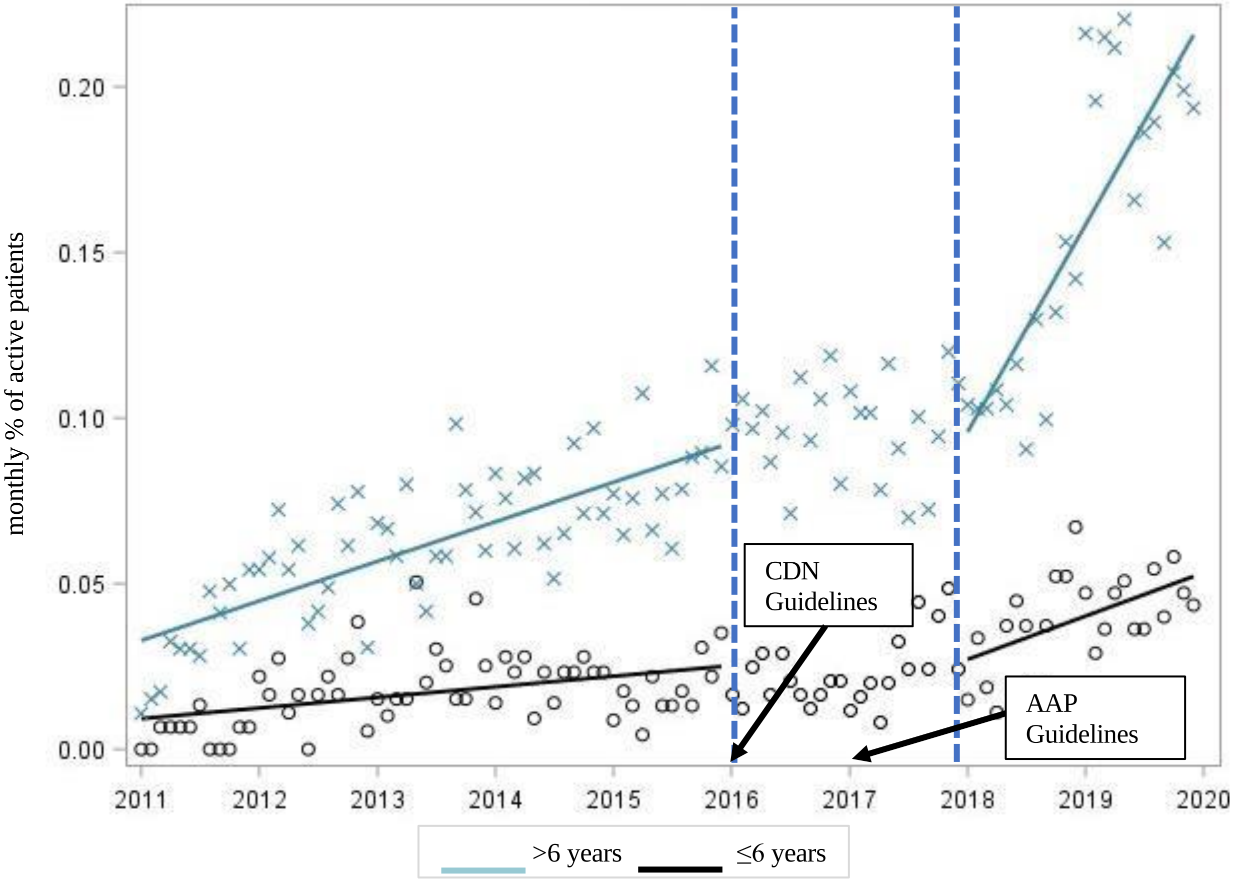

**CDN Guidelines: Hypertension Canada guidelines*
**AAP Guidelines: American Academy of Pediatrics Guidelines*

© 2024 Wu M et al. *JAMA Netw Open.*

eFigure 10. The Proportion of Children Who Underwent Laboratory Testing or Had a Medication Follow-Up From January 1, 2011 to December 31, 2019 by a Primary Care Provider

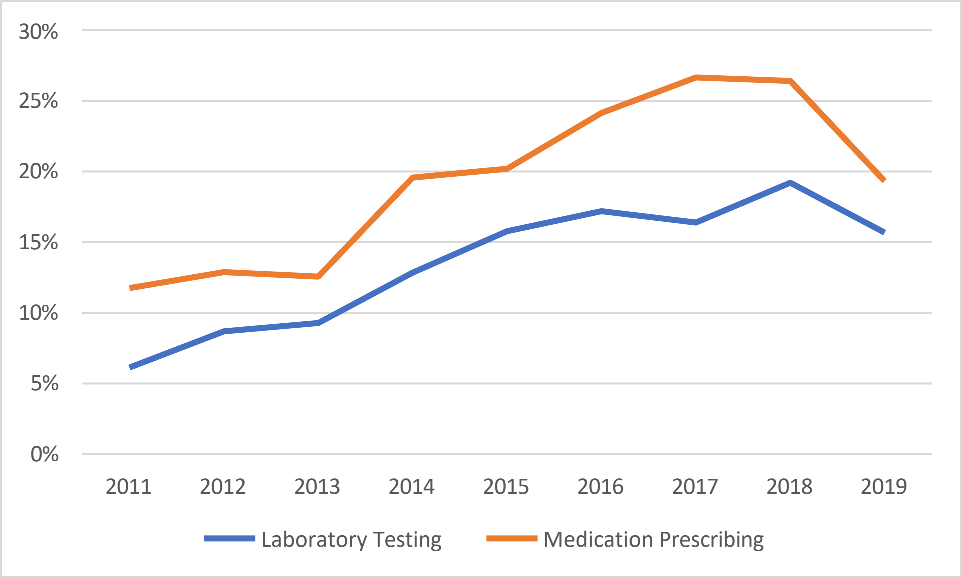

© 2024 Wu M et al. *JAMA Netw Open.*
