# Supplementary material for: Temporal Trends in Practice Patterns After Introduction of Pediatric Hypertension Guidelines in Canada
Source: JAMA Netw Open. 2024 Feb 8;7(2):e2355239. doi: 10.1001/jamanetworkopen.2023.55239 (PMC10853834; doi:10.1001/jamanetworkopen.2023.55239)
Supplement: Supplement 2. — Data Sharing Statement [file jamanetwopen-e2355239-s002.pdf]

Data Sharing Statement

Wu. Temporal Trends in Practice Patterns After Introduction of Pediatric Hypertension Guidelines in Canada. *JAMA Netw Open*. Published February 08, 2024.
doi:10.1001/jamanetworkopen.2023.55239

Data
Data available: No
